# Self-Serving Bias in Performance Goal Achievement Appraisals: Evidence From Long-Distance Runners

**DOI:** 10.3389/fpsyg.2022.762436

**Published:** 2022-02-10

**Authors:** Moonsup Hyun, Wonsok F. Jee, Christine Wegner, Jeremy S. Jordan, James Du, Taeyeon Oh

**Affiliations:** ^1^Department of Business and Economics, Utica College, Utica, NY, United States; ^2^School of Marketing, Entrepreneurship, Sport Management, Hospitality and Tourism, Western Carolina University, Cullowhee, NC, United States; ^3^Department of Tourism, Recreation and Sport Management, University of Florida, Gainesville, FL, United States; ^4^School of Sport, Tourism and Hospitality Management, Temple University, Philadelphia, PA, United States; ^5^Department of Sport Management, Florida State University, Tallahassee, FL, United States; ^6^Department of Health, Exercise Science, and Recreation Management, University of Mississippi, Oxford, MS, United States

**Keywords:** self-serving bias, athletic performance, goal achievement, event satisfaction, participant sport events

## Abstract

While working with a long-distance running event organizer, the authors of this study observed considerable differences between event participants’ official finish time (i.e., bib time) and their self-reported finish time in the post-event survey. Drawing on the notion of self-serving bias, we aim to explore the source of this disparity and how such psychological bias influences participants’ event experience at long-distance running events. Using evidence of 1,320 marathon runners, we demonstrated how people are more likely to be subject to a biased self-assessment contingent upon achieving their best finish time at the event. The study samples were split into record-high-achieved and record-high-missed groups, and the self-serving biases of each group were explored. Results from the *t*-test comparing record-high-achieved and -missed groups showed that runners in the record-high-missed group were significantly more likely to report a positively biased finish time than runners in the record-high-achieved group (*p* < 0.01). Additionally, results from logistic regression showed that as runners missed their best finish time by a wider margin, the probability of reporting a positively biased incorrect finish time increased. Lastly, we conducted an additional *t*-test and revealed that runners who are subject to self-serving bias showed a lower level of overall event satisfaction. The current study suggests one way to bypass the adverse effects of participant sport event participants’ worse-than-expected athletic performance. We specifically suggest that the event organizers target runners who had worse-than-expected performance and make extra efforts on non-race service attributes (e.g., finish line experience, rest and recovery area, and transportation after the event) because these runners are more likely to be unsatisfied with the event.

## Introduction

Competition is often considered a key element of sports (Anderson-Butcher et al., 2014). The outcome of competition in sport contexts can greatly influence fans’ and recreational athletes’ experiences (Yoshida and James, 2011; Du et al., 2015). For instance, competition outcomes in a spectator sport services context—who wins and who loses—have been identified as a core element of service quality evaluation (Greenwell et al., 2002; Theodorakis et al., 2013). Scholars studying participant sport services have likewise found that athletic performance in participant sport events is a critical antecedent of participants’ evaluations of their overall event experiences (Du et al., 2015; Hyun and Jordan, 2020). For instance, failing to achieve a pre-determined athletic performance goal in a long-distance running event results in negative perceptions of the entire event (Hyun and Jordan, 2020). Conversely, runners who achieve their pre-determined goals tend to be more satisfied with the entirety of the event journey even if other service aspects were disappointing (Du et al., 2015).

To better understand effective managerial responses to long-distance running event participants’ behavior, one would be remiss to simply conclude that undesirable competition outcomes will lead to lower levels of satisfaction (e.g., if sport participants cannot achieve their athletic goals in a participant sport event, whey will not be satisfied with the event). If this were the case, there would be little investment that managers can make to change the experience of participants. However, when people encounter negative outcomes, they do not remain unsatisfied but instead seek to alleviate negative emotions—or mitigate the experiences tied to negative outcomes—by using various coping strategies (Snyder et al., 1986; Poczwardowski and Conroy, 2002). In better understanding how to identify these strategies, managers may better be able to understand how to interpret and act upon event feedback from participants.

Per attribution theory (Kelley, 1973), self-serving bias is one of the effective coping strategies that can protect one’s self-esteem and alleviate negative emotions from undesirable outcomes (Kashima and Triandis, 1986; Zhang et al., 2018). People tend to take credit for positive outcomes while attributing negative outcomes to external factors; therefore, people are less likely to be satisfied with external factors when the outcome is negative or less than their expectations. For instance, collegiate Division 1 wrestlers tend to attribute their win to more internally caused and internally controlled factors (e.g., one’s own athletic ability) compared to losers (De Michele et al., 1998). Self-serving bias is also observed in long-distance running event settings: Runners tend to over-credit their ability in a rosy manner after the event even if they fail to meet their preset time goal (Lemm and Wirtz, 2013). In addition, runners who are not satisfied with their athletic performance are also likely to be unsatisfied with the overall event (Du et al., 2015; Hyun and Jordan, 2020).

The purpose of this study is to understand how long-distance running event participants appraise their own athletic performance after the event and how runners with undesirable athletic performance cope with dissatisfaction with their performance. In particular, while working with a long-distance running event organizer for their participant experience analysis, the authors observed considerable differences between runners’ official finish time (i.e., bib time) and their self-reported finish time in the post-event survey. Therefore, in the current study, drawing on the concept of self-serving bias, we seek to answer the following research question: what drives the difference between official finish time and self-reported finish time of long-distance running event participants, and what implications do this long-distance runners’ behavior have for participant sport event organizations?

The survey utilized in this study was distributed to the potential respondents with a clear note that the research team was collaborating with the marathon event organization to understand event participants’ behavior. As such, the respondents were very likely to know that the survey administrators have information about their official finish time (i.e., bib time), while many previous studies in the similar contexts were based on surveys that respondents answered survey questions with being unsure whether the survey administrators had the objective measure of the questions in the surveys (e.g., Gil and Mora, 2011; Markle et al., 2018; Flegal et al., 2019). It would be a strength of the current study that this is a clear test of self-serving bias because it examines whether runners misreport their finish time (i.e., self-serving bias) although they might know that the survey administrators could have information about their official time performance. As such, it would be a novel and effective approach to test self-serving bias in the context of participant sport events where the athletic performance (i.e., marathon finish time) is very important to participants, but there are no prominent rewards of successful performance.

### Performance Appraisal in Participant Sport Events and Coping Behavior

Irrespective of spectator or participant sport contexts, competition appraisals, often in the forms of athletic performance assessment, are contingent upon an individual’s ability to set realistic and rational expectations. In particular, a marathon is a highly goal-directed sport event for amateur runners (Lemm and Wirtz, 2013). For instance, runners participating in a long-distance running event often cite goal achievement as a motivation; they try to run faster and beat a specific time goal, and improve their record-high finish time (Funk et al., 2011; Lemm and Wirtz, 2013). As such, in a long-distance running event, participants often appraise their athletic performance using a certain finish time as a reference point (Markle et al., 2018). The achievement of this referenced finish time has a great effect on a runner’s experience with an event. Many sport management studies in the domain of participant sport have discussed the appraisal of athletic performance goal achievement and its ramifications for contestants’ experience at participant sport events (e.g., perceived service quality, event satisfaction, and re-participation intention; Lemm and Wirtz, 2013; Du et al., 2015; Markle et al., 2018; Hyun and Jordan, 2020).

In particular, a marathon is a race against the clock for amateur runners participating in a long-distance running event. Amateur runners care little about their ranking among participants; rather, they pay special attention to their best finish time (i.e., personal improvement referred in the concept of mastery goal; Adie et al., 2008). In other words, a runner’s best finish time is very likely to be a strong self-referential criterion (i.e., reference point) of their athletic performance appraisal in a long-distance running event. Interestingly, prospect theory suggests that the marginal utility of fulfilling the reference point as opposed to performance failure might not follow a linear continuum (Kahneman and Tversky, 1979). That is, the evaluation of goal achievement is characterized by a reference-dependent S-shaped value function where there is a huge non-linear gap in utility between people whose performance is just below a referenced finish time (i.e., goal missed) and people whose performance is just above a reference point (i.e., goal achieved; Allen et al., 2016).

#### Effects of Athletic Performance in Long-Distance Running Events

According to Du et al. (2015), achieving an athletic performance goal (i.e., breaking a referenced finish time) in participant sport events are sometimes strong enough to compensate for negative experiences involving service provider–generated factors; people with a high level of goal achievement maintained high overall event satisfaction regardless of other factors. For instance, service quality, which is often suggested as one of the most important predictors of consumers’ satisfaction with service products, became powerless in predicting event satisfaction for runners who achieved their athletic performance goals. By contrast, service quality and overall event satisfaction had a strong linear relationship for runners who missed their pre-determined athletic goals: as service quality evaluations increased, so did overall event satisfaction (Du et al., 2015). This finding offers critical insight into the role of athletic goal achievement in participant sport event contexts: athletic goal achievement is such a pivotal aspect of the participant sport event experience that it exerts a strong impact on runners’ overall experience with a participant sport event and may determine runners’ post-event evaluation of the event and behavior.

#### Self-Serving Bias in Coping With Performance Failure

##### Self-Serving Bias

Self-serving bias is derived from a large body of evidence from behavioral economics and cognitive psychology (Gilovich et al., 2002), which refers to humans’ tendency to attribute positive outcomes to their own abilities but ascribe negative outcomes to external factors (Campbell and Sedikides, 1999). This psychological assessment can be triggered by the notion of cognitive dissonance in which individuals subconsciously and inadvertently suppress noise that conflicts with pre-determined expectations of athletic performance (Kelley, 1973; Campbell and Sedikides, 1999; Gilovich et al., 2002). Self-serving bias is often observed in the context where an individual’s self-esteem is being threatened by negative outcomes of one’s effort (Blaine and Crocker, 1993), and this is what usually happens in a marathon event when a runner’s finish time is worse than their expectations. For example, in the context of a marathon event, it has been shown that runners’ worse-than-expected finish time led to many negative consequences, such as a decreased level of event satisfaction, re-participation intention, and future exercise intention (Lemm and Wirtz, 2013; Du et al., 2015; Hyun and Jordan, 2020). As achieving a good time performance is one of the critical factors affecting runners’ post-event perception and behavior, runners whose finish time is worse than their best time are likely to be affected by self-serving bias.

Self-serving bias can be exhibited in many different forms of behavior, and one of which is conscious or unconscious dishonest behavior (Mazar and Ariely, 2006). As per the theory of self-deception (Trivers, 2000), self-serving bias (i.e., a strong desire to improve or maintain self-esteem) could lead to dishonest behavior even when there are no external benefits of being dishonest; self-deception represents “a biased, self-serving information flow within an individual—that is, an active but unconscious misrepresentation of reality to the conscious mind” (Mazar and Ariely, 2006, p. 122). Self-deception allows individuals to maintain a positive sense of self in the wake of moments of confusion, frustration, dissatisfaction, or other emotions that arise from negative outcomes. As noted above, participants of long-distance running events would feel a deep frustration when their athletic performance is worse than their expectations. To avoid or alleviate this frustration and maintain their self-esteem, they would have self-serving bias (i.e., ascribing their failure to external factors), which would lead them to have a biased, self-serving information about their athletic performance. As such, we hypothesize that long-distance runners whose finish time is worse than their best time performance will be likely to report a positively biased finish time (i.e., self-reported finish time that is faster than official finish time)—even in a situation with no apparent benefits of being dishonest, such as an anonymized survey.

*H_1_: Long-distance runners whose official finish time is slower than their best finish time will exhibit a stronger self-serving bias compared to runners whose official finish time is faster than their best finish time.*

*H_1*a*_: The former will more frequently exhibit a self-serving bias than the latter.*

*H_1*b*_: The former will more strongly exhibit a self-serving bias than the latter.*

*H_2_: As the extent to which a long-distance running event participant’s time performance falls short of their best finish time increases, the probability of them reporting a positively biased finish time will increase.*

At the same time, long-distance runners who are subject to self-serving bias will be less likely to be satisfied with the overall event because they tend to blame the external factors and event environment for their failure. This tendency would be particularly salient among runners who reported a positively biased self-reported finish time because self-deception is evidence that they were actively utilizing self-serving bias as a coping strategy to avoid any negative emotion from the failure. Runners who are subject to self-serving bias would believe that the event environment was the cause of their poor athletic performance; for example, they might think that the course was too difficult, weather was not supportive, water station was inconvenient, time markers were hard to read, there were too many participants, or shoes were uncomfortable. As such, we hypothesize that athletes who report positively biased incorrect time performance in a post-event anonymous survey (i.e., those who actively used a coping strategy to protect their self-esteem due to self-serving bias) will have a low level of overall event satisfaction compared to runners who did not use a coping strategy (i.e., those who reported a correct finish time in a post-event anonymous survey).

*H_3_: Long-distance runners whose self-reported finish time is positively biased (i.e., better than their official finish time) will have a significantly lower level of overall event satisfaction compared to runners who correctly reported their finish time in the post-event anonymous survey.*

## Materials and Methods

### Study Participants

To test the proposed hypotheses, we conducted a series of statistical analyses based on the official time performance database and post-event survey from a long-distance running event held in the southern United States in 2016. The event included 23,001 participants among full marathon, half marathon, and 5K runners. Among them, 1,320 participated in the current study. Demographically, 44.17% of the current sample were women, 46.82% were Hispanic, 44.24% were Caucasian, 84.24% held at least a 4-year college degree, and 53.41% earned an annual household income greater than $80,000. Athletes’ average age was 40.39 (*SD* = 10.88), with a median age of 40. We also compared survey respondents’ age and gender to those of all registered runners. The average age of all event participants was 39.52 (*SD* = 10.78) with a median age of 39; 49.30% were women. Therefore, survey respondents represented the entire sample well in terms of age and gender, despite a response rate of 15.56%. Sample characteristics are summarized in Table 1.

**TABLE 1:** Sample characteristics.

Sample characteristics	Percentage
**Gender**	
Male	53.2%
Female	46.8%
**Ethnicity**	
Hispanic	46.8%
Caucasian	44.2%
African American	4.0%
Asian	1.7%
Native American	0.1%
Pacific Islander	0.1%
**Education**	
Less than 4-year degree	15.8%
4-year or postgraduate degree	84.2%
**Annual household income**	
<$80,000	46.6%
≥$80,000	53.4%
**Age**	Mean = 40.39 (*SD* = 10.88)

### Procedure

The data were collected from three sources. First, when registering for the event, participants were required to provide their best finish time with the purpose of assigning an appropriate starting corral. Second, every participant’s official finish time was measured by a digitalized system (i.e., bib time) by the event organizer. Third, a post-event survey was emailed to all participants a week after the event using their email addresses in the event organizer’s race registration database. The post-event survey was designed to measure long-distance running event participants’ athletic performance appraisals and event satisfaction; therefore, the survey included questions about their race finish time, event satisfaction, and a group of control variables (i.e., running involvement, past running experience, intensity and frequency of daily exercise, and demographics; see Table 2).

**TABLE 2:** Post-event survey measurement items.

Construct	Item
Self-reported finish time	What was your actual time for the event?
Event satisfaction (α = 0.80)	I was satisfied with my decision to participate in this event
	I did the right thing by deciding to participate in this event
	I was happy that I decided to participate in this event
Running involvement (Sign) (α = 0.84)	Running says a lot about who I am Running tells something about me
	Running gives others a glimpse of the type of person I am
Past experience	Not including this event, how many organized running events have you registered for and participated in during the last 12 months?
Intensity of daily exercise	In general, how many miles per week do you run as part of your physical activity?
Frequency of daily exercise	In general, how many days per week do you spend running as part of your physical activity?

Among the 23,001 participants, 3,579 completed the survey (response rate: 15.56%). After the survey data were collected, respondents’ best finish time and official finish time were added to the dataset. 5K runners were excluded from the dataset because shorter distance runners are likely to be inexperienced runners and have a lower commitment to running compared to half and full marathon participants (Funk et al., 2011), resulting in being less likely to care about their finish time. In addition, seasoned runners who are participating in half or full marathon often participate in 5K as a rehearsal (5K is usually held a day before half/full marathon). In this event, approximately 53.8% of 5K runners also participated in half/full marathon. Also, runners who reported that finish time is not of their interest (i.e., no finish time goal) were excluded from the dataset. Ultimately, 1,320 survey responses were matched based on valid best finish time in the race registration, a self-reported finish time in the survey responses, and an official finish time in the performance database. The data collected from the same event but in a different year were used by the research team in an already published study. In the current study, we investigated whether there were any repeat participants from our prior dataset. We found that 9.9% of respondents in this study were also included in our previous data. These respondents were retained in the present dataset because the purpose of this study is distinct from our earlier work (Fine and Kurdek, 1994).

### Experimental Parameters

Self-reported finish time was measured in the post-event survey by asking “What was your actual time for the event?” Overall event satisfaction was assessed using a 3-item construct adapted from Oliver (1980), scored on a 7-point Likert scale; for instance, “I was satisfied with my decision to participate in this event” (1 = *strongly disagree*, 7 = *strongly agree*). Then, the average score on the three items was calculated to analyze the data. Athletic performance was measured by comparing participants’ referenced finish time (i.e., the best finish time in the past races) and official finish time recorded by the digitalized system (Du et al., 2015; Markle et al., 2018). The difference between the two finish times was calculated by subtracting each participant’s official finish time from their best finish time. Self-serving bias was evaluated by the discrepancy between the self-reported ability of oneself or achievement and objectively measured performance in a certain task (Alicke et al., 1995; Trivers, 2000; Mazar and Ariely, 2006). Specifically, in the current study, participants’ self-reported finish time and official finish time (i.e., bib time) were compared, and the magnitude of self-serving bias was calculated by subtracting each participant’s self-reported finish time from their official finish time. For example, if a runner’s official finish time was 1 h 50 min and their self-reported finish time was 1 h 40 min, then the magnitude of self-serving bias was 10 min.

After data cleaning, two dummy variables were created: (1) record-high-achieved and record-high-missed groups and (2) biased and unbiased groups. First, based on the athletic performance measure, study participants were classified into either the record-high-achieved or record-high-missed group. Specifically, if the measure of athletic performance was positive (i.e., the official finish time was faster than the best finish time), then a runner was included in the record-high-achieved group. Conversely, if the gap between best finish time and official finish time (i.e., athletic performance measure) was negative (i.e., the official finish time was slower than the best finish time), runners were categorized into the record-high-missed group. Ultimately, 635 runners fell into the record-high-achieved group, and 685 fell into the record-high-missed group.

Second, respondents were classified into either the biased or unbiased group based on the self-serving bias measure (i.e., the gap between self-reported time performance and official time performance). If the self-serving bias measure was greater than 59 s (i.e., self-reported time was faster than official finish time by more than 59 s), a runner was deemed as having misreported their time performance and was placed in the biased group. A difference of less than 1 min was not considered deception because self-reported finish time was collected in only hour(s) and minute(s), whereas official finish times included second(s), and marathon event participants largely tended to ignore seconds when discussing their time performance (i.e., rounding down). All other runners whose self-serving bias measure was less than 1 min (i.e., the discrepancy between self-reported time performance and official time performance was less than 1 min) were included in the unbiased group. Consequently, 161 runners were placed in the biased group while 1,159 were placed in the unbiased group.

#### Control Variables

Several variables that might influence runners’ self-serving bias were included as control variables. In this study, we hypothesized that self-serving bias would be used as a coping strategy in stressful situations where an athlete’s positive self-image as a runner is threatened (Taylor and Armor, 1996). Therefore, variables that could represent runners’ involvement in the sport were included because they were likely to be strongly associated with study participants’ self-image as a runner. First, among three sub-dimensions of the running involvement construct (i.e., *sign*, *centrality*, and *pleasure*), *sign* was included because it represents the self-expression value, or symbolism, of an activity (running, in our case, such as indicated by the statement “Running says a lot about who I am”; Beaton et al., 2011). *Centrality* and *pleasure* were not included because they reflect the importance of running and the extent to which people enjoy running in daily life rather than one’s self-image as a runner. Second, participants’ behavioral representations of involvement in running were included as control variables: past experience (the number of events in which they had participated), intensity of running as a form of exercise (“In general, how many miles per week do you run as a part of your physical activity?”), and frequency of running as a form of exercise (“In general, how many days per week do you spend on running as a part of your physical activity?”). Summary statistics appear in Table 3.

**TABLE 3:** Descriptive statistics of focal and control variables.

	Full sample (*N* = 1,320)		Record-high achieved (*N* = 635)		Record-high missed (*N* = 685)	
	*M*	*SD*	*M*	*SD*	*M*	*SD*
**Focal variables**						
Athletic performance (record-high – official finish time)	−1.23	26.95	14.91	25.39	−16.20	18.35
Self-serving bias (official – self-reported)	1.47	5.08	0.81	2.42	2.08	6.60
Event satisfaction	6.44	0.71	6.52	0.64	6.37	0.77
**Control variables**						
Running involvement (Sign)	5.87	0.99	5.89	0.98	5.86	1.00
Past experience	6.43	23.05	6.54	32.43	6.33	7.05
Intensity of daily exercise	22.22	13.30	22.02	12.73	22.41	13.82
Frequency of daily exercise	3.86	1.28	3.88	1.26	3.85	1.30

### Data Analysis

**FIGURE 1:**
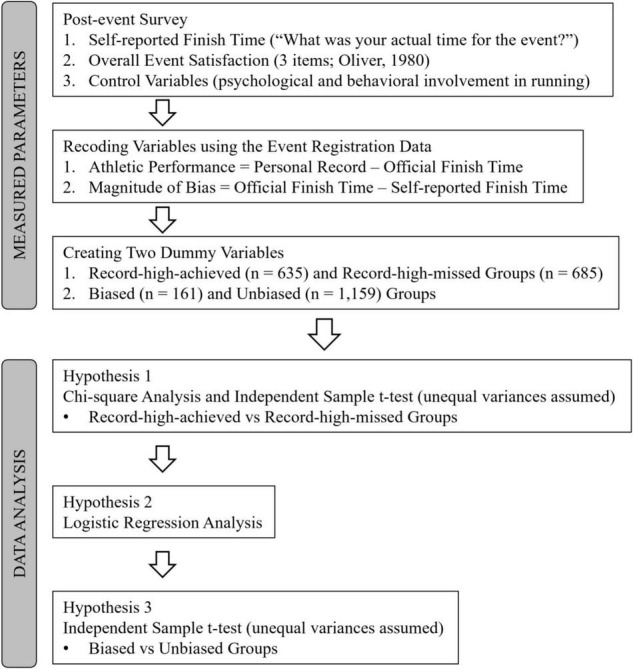
Flow chart for the testing process and measured parameters.

All statistical analyses were performed in R 3.6.2 and SPSS 27. First, to test Hypothesis 1, a chi-square analysis and an independent sample *t*-test with the assumption of unequal variances were conducted. In these tests, the magnitude of self-serving bias between the record-high-achieved and record-high-missed groups was statistically compared to determine whether runners in the record-high-missed group reported a positively biased time performance more frequently and strongly than those in the record-high-achieved group. Second, logistic regression analysis was used to test Hypothesis 2; we tested whether the magnitude of missed record-high finish time (i.e., the difference between one’s best finish time and official finish time) affected the probability of runners reporting a positively biased self-reported finish time. Last, to test Hypothesis 3, another independent sample *t*-test was conducted with the assumption of unequal variances. In this test, the overall event satisfaction between the biased and unbiased groups was statistically compared to examine whether runners in the biased group reported a lower level of overall event satisfaction than those in the unbiased group. A 5% significance level was used in all the statistical tests. The data analysis process is depicted in Figure 1.

## Results

### Frequency and Magnitude of Self-Serving Bias

As shown in Table 4, the chi-square analysis indicated that the frequency of reporting a positively biased finish time in a post-event survey varies significantly based on whether runners achieved their record-high or not [*x*^2^ (1) = 48.69, *p* < 0.01]. Specifically, for the record-high missed group, the number of runners who reported a positively biased finish time was significantly more than expected (*p* < 0.01); 125 out of 685 runners (18.2%) reported a positively biased finish time. On the contrary, the number of runners who reported a positively biased finish time was significantly fewer than expected in the record-high achieved group (*p* < 0.01); only 36 out of 635 runners (5.7%) reported a positively biased finish time. These results supported Hypothesis 1a.

**TABLE 4:** Results of chi-square test.

	Record-high missed vs. achieved		
	Missed group	Achieved group	Full sample
**Biased group**			
Count	125 (18.2%)	36 (5.7%)	161 (12.2%)
Expected count	83.5	77.5	161
Adjusted residual (*p*-value)	4.5 (<0.01)	−4.7 (<0.01)	
**Unbiased group**			
Count	560 (81.8%)	599 (94.3%)	1,159 (87.8%)
Expected count	601.5	557.5	1,159
Adjusted residual (*p*-value)	−1.7 (>0.05)	1.8 (>0.05)	
Total	685	635	1,320 (100%)

In the independent sample *t*-test, the level of self-serving bias (i.e., the difference between one’s official time performance and self-reported time performance) was compared between the record-high-achieved and record-high-missed groups. We observed a significant difference in the level of self-serving bias between the two groups. The record-high-achieved group’s average extent of self-serving bias was 0.81 min (*SD* = 2.42, *n* = 635), equal to roughly 49 s. For example, if a runner self-reported her/his finish time as 1 h 50 min, then the official finish time was 1 h 50 min 49 s, on average. Essentially, the record-high-achieved group rarely reported their time incorrectly except for a rounding pattern that ignored seconds. On the contrary, the average level of self-serving bias in the record-high-missed group was 2.08 min (*SD* = 6.60, *n* = 685), which is about 2 min 5 s; that is, the record-high-missed group consciously or unconsciously reported time performance incorrectly by an average of 2 min 5 s. The *t*-test results, summarized in Table 5, revealed that this mean difference was statistically significant (*t* = −4.69, *df* = 877.49, *p* < 0.05), supporting Hypothesis 1b.

**TABLE 5:** Results of independent sample *t*-test with unequal variance assumption.

	Full sample		Record-high achieved (*N* = 635)		Record-high missed (*N* = 685)		*t*	*df*	*p*-value
	*M*	*SD*	*M*	*SD*	*M*	*SD*			
Self-serving bias (official – self-reported) (Hypothesis 1a)	1.47	5.08	0.81	2.42	2.08	6.60	−4.69	877.49	<0.01

### Probability of Misreporting Finish Time

**FIGURE 2:**
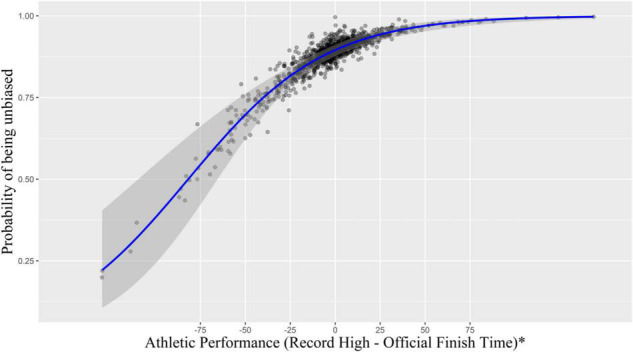
Probability of being unbiased based on the level of athletic performance. *A positive number of athletic performance indicates that a runner broke her/his record-high at the event. The solid blue line shows predicted effects of Athletic Performance, the gray area shows 95% CI, and the black dots represent the actual data values.

Logistic regression analysis was used to test Hypothesis 2. A dummy variable was created using a self-serving bias measure (0 = biased, 1 = unbiased) and was regressed on the level of athletic performance (i.e., record-high – official finish time); a group of control variables was also included in the model (i.e., frequency of physical activity, intensity of physical activity, past event experience, and running involvement). Because the athletic performance was coded based on best finish time minus official finish time, the level of athletic performance increased as a runner’s official finish time was faster than her/his best finish time by a wider margin. According to the results in Table 6, a significant association appeared between one’s level of athletic performance and self-serving bias, indicating that failure to beat one’s best finish time led to a higher probability of being categorized into the biased group [β = 0.701, exp(β) = 2.015, *p* < 0.05]. Thus, Hypothesis 2 was supported. The pseudo *R*^2^ (i.e., Nagelkerke *R*^2^) of the model was 0.09. Based on the logistic regression results, each runner’s probability of reporting a positively biased self-reported finish time was estimated given the level of athletic performance, as shown in Figure 2.

**TABLE 6:** Estimates from logistic regression for self-serving bias.

Variables	β	exp(β)	*Z*-value
Control variables			
Running involvement (Sign)	0.050	1.052	0.579
Past experience	−0.003	0.997	−0.036
Intensity of daily exercise	0.290*	1.336	2.198
Frequency of daily exercise	−0.173	0.841	−1.512
Athletic performance (Hypothesis 1b)	0.701*	2.015	7.017
Pseudo *R*^2^ (Nagelkerke) = 0.09			

*Dependent variable: dummy for self-serving bias (0 = biased group, 1 = unbiased group).*

*p < 0.05.

### Effects of Self-Serving Bias on Event Satisfaction

To test Hypothesis 3, an independent sample *t*-test was performed. According to the results, the level of overall event satisfaction was significantly different between the biased group and the unbiased group. The biased group’s average overall event satisfaction was 6.31 (*SD* = 0.83, *n* = 161), while the unbiased group’s average overall event satisfaction was 6.46 (*SD* = 0.69, *n* = 1,159). The *t*-test results, summarized in Table 7, indicated that the mean difference between the two groups was statistically significant (*t* = −2.13, *df* = 192.02, *p* < 0.05), supporting Hypothesis 3.

**TABLE 7:** Results of independent sample *t*-test with unequal variance assumption.

	Full sample		Biased group (*N* = 161)		Unbiased group (*N* = 1,159)		*t*	*df*	*p*-value
	*M*	*SD*	*M*	*SD*	*M*	*SD*			
Overall event satisfaction (Hypothesis 2)	6.44	0.71	6.31	0.83	6.46	0.69	−2.13	192.02	<0.05

## Discussion

This study enriches understanding of recreational athletes’ behavior in a participant sport services context by examining how these runners sought to cope with less-than-expected athletic performance. In response to our research question, self-serving bias was hypothesized as a coping strategy among runners who missed their best finish time at a long-distance running event. The hypotheses were developed based on the concept of self-serving bias, which posits that people tend to have positively biased perception toward oneself by ascribing failures to external factors to overcome dissatisfaction with one’s capability to complete a certain task (Gilovich et al., 2002). Above all, our findings aligned with the notion of self-serving bias; runners whose finish time fell short of their best finish time were more likely to report positively biased incorrect finish time than those whose time performance was better than their best finish time, despite the lack of external reward for doing so. As suggested by studies regarding self-serving bias, such behavior could be interpreted as a means of coping with potential dissatisfaction or discomfort with one’s less-than-expected athletic performance at the event; participants might have deceived themselves in an effort to protect their positive self-image as a runner (Campbell and Sedikides, 1999; Trivers, 2000; Mazar and Ariely, 2006).

Specifically, a significant positive relationship was found between athletic performance and the probability of self-reporting a positively biased finish time: As long-distance running event participants failed to break their best finish time by a wider margin, the probability of reporting positively biased incorrect finish time increased (see Figure 2). As illustrated, if a runner missed their goal by approximately 20 min, the probability of deception (i.e., self-reporting positively biased incorrect time performance) was roughly 20.00%. Comparatively, if a runner achieved their goal by approximately 20 min, the likelihood of misreporting their finish time was only 8.12%; there was an 11.88 percent points increase in the probability of misreporting between the two. This result might be surprising to event organizers because runners had no explicit reason to misreport their finish time: they would receive no external benefits or rewards for reporting better time performance in an anonymous survey.

Many researchers have found similar biases in several different contexts; people tend to have positive views of themselves with the purpose of maintaining their self-image (Taylor and Armor, 1996; Trivers, 2000; Mazar and Ariely, 2006). For instance, people tend to have a positively biased perception toward their physical self-image (i.e., body image). Flegal et al. (2019) showed that, regardless of gender, self-reported height was significantly higher than measured height based on the National Health and Nutrition Examination Survey collected in the United States. Gil and Mora (2011) also identified similar bias in reporting height and weight using Health Examination Surveys conducted in Spain; as individuals’ satisfaction with their own body image increases, the probability of misreporting their weight decreases. Furthermore, people frequently overestimate their own qualities and abilities and believe that they are better than their average peer (i.e., better-than-average effect; Alicke et al., 1995; Alicke and Govorun, 2005).

The underlying reasons on why people exhibit these biases in various contexts could be largely explained by self-determination theory (Ryan and Deci, 2019). Self-determination theory is comprised of six “mini-theories,” and one of which is basic psychological needs theory (Ryan and Deci, 2019). According to basic psychological needs theory, people have three fundamental psychological needs, namely autonomy (feeling a sense of choice about one’s behavior), competence (being able to bring about positive changes in desired outcomes), and relatedness (feeling accepted by one’s social milieu; Ntoumanis et al., 2021). The frustrations (and satisfactions) of these needs would impact one’s wellness and optimal functioning (Ryan and Deci, 2019), resulting in amotivation, depression, and unhealthy behavior when frustrated (Ng et al., 2013; Ntoumanis et al., 2021) and lower amotivation and adaptive behavior when satisfied (Vansteenkiste et al., 2018; Ntoumanis et al., 2021; Pope et al., 2021). In the current study, runners who experienced performance failure were likely to encounter frustration in their self-determined motivations, such as competence and relatedness, because they failed to achieve their goals despite the significant amount of time and effort they put into the preparation for the event. To protect their self-image and prevent any negative emotion resulting from such frustration, runners would be inclined to cope with their less-than-expected athletic performance by self-serving bias (Campbell and Sedikides, 1999).

Our findings also imply that self-serving bias affects event participants’ overall perceived event experience. Specifically, a significant difference in overall event satisfaction between the biased and unbiased groups was found in this study. Per prior research regarding self-serving bias (Campbell and Sedikides, 1999), people who experienced a failure in a certain task tend to ascribe their failure to external factors. As such, they are less likely to be satisfied with the surrounding environment. In the context of long-distance running events, runners whose athletic performance is worse than their expectation would blame the event environment, rather than their own ability, for their worse-than-expected athletic performance. As suggested in self-serving bias literature (De Michele et al., 1998), this would lead them to believe that the event environment prevents them from achieving their record-high finish time. Conceptually, reporting a positively biased incorrect finish time in an anonymous survey shows that a runner is subject to self-serving bias. Therefore, as shown in this study, they are less satisfied with the overall event compared to runners who are not subject to self-serving bias; runners exhibiting self-serving bias are very likely to think that their worse-than-expected finish time was due, in part, to the event environment.

Theoretically, this study underlines self-serving bias as one way recreational athletes handle disappointing competition outcomes in a participant sport services context. Unlike spectator sport, scholars cannot and do not consider “reflected” glory or “reflected” failure (i.e., BIRGing and CORFing; Cialdini et al., 1976; Campbell et al., 2004) in participant sport services because competitions are completed by athletes themselves. That is, both glory and failure in competitions at participant sport events are tied to athletes’ own outcomes. This nature of participant sport thus requires a unique approach to understanding participant sport consumers’ strategies for coping with disappointing competition outcomes. The current study thus offers a new theoretical perspective on participant sport consumers’ coping behavior in a participant sport events context. Specifically, we propose self-serving bias as a potential coping strategy for personal failure in achieving athletic performance goals in a long-distance running event; by engaging in self-serving bias, runners are cutting off performance failure (COPFing).

### Practical Implications

With regard to athletic performance and self-deception as a coping strategy, the current study has some important practical takeaways. Although the current study suggests that runners whose athletic performance is worse than their best finish time are likely to report a positively biased finish time, such bias neither harms anybody nor has undeserved external benefits. People merely try to protect their self-esteem from their own failure in sport competitions (e.g., failing to break record-high performance). However, participant sport event organizers should understand this process of how event participants cope with their worse-than-expected athletic performance. In other words, they must be careful in how they internalize event feedback. Researchers in spectator sport have cautioned about the differences in event experience feedback after a win versus a loss (Matsuoka et al., 2003). In participant sport events, there are always going to be those who are satisfied with their personal performance and those who are dissatisfied. When participants fail to achieve their goal, they blame the event environment for their failure, and these people may have significantly lower event satisfaction; we showed in this study that people who had self-serving bias (i.e., reporting a positively biased finish time) showed a significantly lower level of overall event satisfaction. Identifying the participants subject to self-serving bias allows event managers to understand event feedback more specifically (i.e., those engaging in self-serving bias will be more likely to rate their event experience lower).

In addition, event organizers must make extra efforts for runners who perform worse than expected at the event because they are susceptible to engaging in self-serving bias, which leads them to be less satisfied with the event. For instance, participants’ non-race experience (e.g., supportive staffs, entertainment on the course, cheer zone, food and beverage, rest and recovery area, and content of goody bag) is also an important component of overall event satisfaction and could offset the negative effect of worse-than-expected athletic performance on event satisfaction (Du et al., 2015); it has been shown that there is a high positive association between service quality and event satisfaction among runners who are not satisfied with their athletic performance while runners who are satisfied with their athletic performance are also satisfied with the overall event regardless of their level of service quality perception (Du et al., 2015). Providing participants with the best non-race experience would be one way to bypass the adverse effects of runners’ self-serving bias derived from worse-than-expected athletic performance, which is otherwise largely out of the control of organizers.

### Limitations and Future Research

The current study has some significant limitations. First, rounding errors may have tempered the reliability of our results. Most sample participants rounded down seconds from their time performance; for instance, if a runner’s finish time was 1 h 40 min 50 s, they often reported their finish time as 1 h 40 min. Mathematically speaking, however, their finish time was closer to 1 h 41 min. We did not consider rounding down to be deception in this study because the rounding phenomenon was observed across all participants regardless of their level of goal achievement. In future work, people’s behavioral patterns regarding rounding down and the psychological rationale behind it could reveal intriguing insight.

Second, according to Mazar et al. (2008), self-deception has a short-term effect; while people can maintain their self-esteem through self-serving bias and self-deception, these conciliatory effects only persist for a short time. In the long term, self-deception may backfire due to overestimated self-evaluation. Therefore, we strongly recommend that scholars investigate the potential negative long-term effects of self-deception in a participant sport services context. It would be especially interesting to explore whether athletes who engage in self-deception can maintain a positive self-image for an extended period by tracing temporal changes in their self-image as a runner. In so doing, researchers could develop a more thorough understanding of the effectiveness of self-deception as a coping strategy for worse-than-expected athletic performance in participant sport events.

Last, an important limitation related to identifying self-deception is that a third party cannot identify whether people are intentionally lying or are merely reporting an incorrect time due to inaccurate recall. Because it was impossible to determine respondents’ intentionality in the current study, we cannot affirmatively say whether athletes who missed their time goals were engaging in unconscious self-deception or overt deception. However, the results of this study revealed that the likelihood of reporting an incorrect finish time was statistically significantly higher for runners who missed their best finish time than for those who achieved their best finish time despite a lack of reward for misreporting. Subsequent research should examine runners’ consciousness in their deception more closely, ideally within an experimental setting, to provide additional support and boundary conditions for our findings.

## Conclusion

Marathon running provided a unique environment to examine runners’ self-serving bias through tapping into individual conscious/unconscious yet harmless self-serving coping mechanisms. Through our analysis, we found that runners whose finish time fell short of their best finish time were more likely to self-report positively biased incorrect finish time than those whose time performance was better than their best finish time. Furthermore, we found that runners who reported positively biased finish time (i.e., those who were subject to self-serving bias) showed significantly lower event satisfaction. In conclusion, participant sport event organizers should understand this psychological process of how event participants cope with their worse-than-expected athletic performance and are encouraged to make extra efforts for runners who performed worse than their expectations because they are susceptible to engage in self-serving bias, which will lead them to be less satisfied with the event.

## Data Availability Statement

The raw data supporting the conclusions of this article will be made available by the authors, without undue reservation.

## Ethics Statement

Ethical review and approval was not required for the study on human participants in accordance with the local legislation and institutional requirements. Written informed consent for participation was not required for this study in accordance with the national legislation and the institutional requirements.

## Author Contributions

MH and JJ contributed to the conception and design of the study. WJ, CW, JD, and TO contributed and wrote sections of the manuscript. All authors contributed to manuscript revision, read, and approved the submitted version.

## Conflict of Interest

The authors declare that the research was conducted in the absence of any commercial or financial relationships that could be construed as a potential conflict of interest.

## Publisher’s Note

All claims expressed in this article are solely those of the authors and do not necessarily represent those of their affiliated organizations, or those of the publisher, the editors and the reviewers. Any product that may be evaluated in this article, or claim that may be made by its manufacturer, is not guaranteed or endorsed by the publisher.
